# Reformed conventional curriculum promoting the professional interest orientation of students of medicine: JENOS

**DOI:** 10.3205/zma001258

**Published:** 2019-10-15

**Authors:** Claudia Ehlers, Nadine Wiesener, Ulf Teichgräber, Orlando Guntinas-Lichius

**Affiliations:** 1 Friedrich Schiller University Jena, Medical Faculty, Dean of Studies, Jena, Germany; 2 Jena University Hospital, Institute for Diagnostic and Interventional Radiology, Jena, Germany; 3 Jena University Hospital, ENT Department, Jena, Germany

**Keywords:** JENa professional interest-Oriented Studies (JENOS), Ambulatory-oriented medicine (AoM), bottom-up strategy, Canadian Medical Education Directions of Specialist (CanMEDS) rolls, Clinic-oriented medicine (KoM), constructive alignment, costs, curriculum, deep learning, evaluations, Flexner model, identification, incentives, interactivity, JUH-specific lecturer and student information system (DOSIS), learning portfolio, longitudinal curriculum, mapping, Master Plan 2020, medical didactic programmes, mentoring, organisational difficulties, performance-based compensation, practical orientation, professional interest orientation, reduction of the curriculum, reform, reinforcement of ambulatory and general medicine, research-oriented Medicine (FoM), resources, scientific orientation, small group modules, student centered learning

## Abstract

**Introduction:** In the last ten years, the medical faculty at Friedrich Schiller University Jena has reformed its traditional curriculum for human medicine. The reformed JENa professional interest-Oriented Studies (JEnaer Neigungs-Orientiertes Studium, JENOS) – with the objective to facilitate career entry through a professional interest-oriented practical approach – emerged due to the stipulation of cost neutrality.

**Methods: **Report on the process sequence of JENOS from the reform idea to implementation: the initial processes, the development and assessment process with accompanying dialogue and dispute of the reform process within the faculty shall be discussed. The 17 objectives of the JENOS reformed traditional curriculum shall be presented and the current level of fulfilment assessed.

**Results: **The structural link of the professional interest-oriented proposals was achieved through the recognition by the “Landesprüfungsamt” (State Examination Board) as elective subjects with 21 semester hours (SH). Feedback and evaluations were conducted using lecturer and student information systems that were implemented in parallel. Eleven of 17 objectives have been achieved, three are still in process and three have not been achieved.

**Discussion:** A professional interest orientation could be achieved through the reform. The weaknesses are found primarily in the links between teaching content. These are currently undergoing a mapping process in order to be optimised.

**Conclusions: **Despite cost neutrality, JENOS is the successful result of reforming the curriculum. The academic reform complied with some requirements for the Master Plan 2020 for Medical Studies in order to be able to implement future changes.

## Zusammenfassung

**Einleitung:** Die Medizinische Fakultät der Friedrich-Schiller-Universität Jena hat in den letzten zehn Jahren ihren Regelstudiengang der Humanmedizin reformiert. Unter der Vorgabe der Kostenneutralität ist der reformierte JEnaer NeigungsOrientierte Studiengang (JENOS) – mit der Zielsetzung den Berufseinstieg durch eine neigungsorientierte Praxisorientierung zu erleichtern – entstanden.

**Methodik: **Bericht über den Prozessverlauf von JENOS von der Reformidee zur Umsetzung: Es werden die anfänglichen Prozesse, der Entwicklungs- und Begutachtungsprozess mit dem Dialog und Disput für den Reformprozess in der Fakultät betrachtet. Die 17 Zielsetzungen des reformierten Regelstudiengangs JENOS werden vorgestellt und deren bisheriger Erfüllungsgrad bewertet.

**Ergebnisse:** Die strukturelle Anbindung der neigungsorientierten Angebote erfolgte über die Anerkennung als Wahlfach mit 21 Semesterwochenstunden (SWS) über das Landesprüfungsamt. Feedbacks und Evaluationen wurden über ein zeitgleich eingeführtes Dozenten- und Studierendeninformationssystem durchgeführt. 11 von 17 Zielsetzungen konnten erreicht werden, drei weitere im Ansatz, und drei konnten nicht erreicht werden.

**Diskussion:** Eine Neigungsorientierung konnte mit der Reform realisiert werden. Die Schwächen beziehen sich in erster Linie auf die Verknüpfung der Lehrinhalte, welche aktuell durch einen Mapping-Prozess optimiert werden.

**Schlussfolgerung: **Trotz Kostenneutralität ist es mit JENOS gelungen, eine Studienreform erfolgreich durchzuführen. Die Studienreform hat partielle Voraussetzungen für den Masterplan Medizinstudium 2020 geschaffen, um künftige Veränderungen umsetzen zu können.

## 1. Introduction

In the last ten years, the Medical Faculty in Jena has reformed its curriculum for human medicine. Especially in the beginning of the process, a literature review focused on reforming a study programme for human medicine was conducted. It became apparent that the study programme should be adjusted to be more practice oriented in order to prepare students to solve practical challenges that arise during patient care [1], [2], [3]. Additionally, students should take more responsibility for their own learning (student centred learning) [4]. It is crucial that students can make informed decisions on an obligatory chosen range of modules for their personal educational focus. It is assumed that they can better develop their individual learning strategies [5]. Student should be encouraged to study more outside of the modules. In order to facilitate self-study, more small group modules should be offered, which provide opportunities for interactive, independent and deep learning, which, for example, allow them to understand principles, solve problems and train soft skills [6]. The result is a reformed traditional curriculum on human medicine with the acronym JENOS, which stands for JENa professional interest-Oriented Studies [https://www.gesetze-im-internet.de/_appro_2002/BJNR240500002.html]. This article presents the sequence of the reform process with original ideas and objectives along with difficulties and problems that arose during its implementation. As the academic reform should be implemented in a cost neutral way, resource problems (in particular administration, space, faculty for several small group modules) needed and need to be overcome.

In order to make space for professional interest-oriented teaching content, the current curriculum had to be analysed and reduced. The reduction of redundancies to create space and its implementation within the curriculum will be a special focus of this article, especially as this process was resisted by the clinics and institutes. Despite difficulties, enough time was found for the professional interest orientation within the elective modules with a scope of 21 SH. 

The findings are based on the end-of-semester evaluations (online evaluation) of each semester from summer semester 2015 to summer semester 2017. Marking follows the school marking system from 1.0-6.0 with 1.0 as a very good result as well as the recommendations of the faculty advisory council in December 2016.

## 2. Project description

### 2.1. Cause of academic reform

Initiating an academic reform is a lengthy process, which should be well prepared for in advance. In Jena, the reform was initiated and guided by three factors, which are described below: 

#### 2.1.1. Recommendations

Recommendations for optimising the study of human medicine at the Jena University Hospital (JUH) were presented during the evaluation by the science council as well as during authorisation from the Integrated Research and Treatment Center (IRT) for Sepsis Control and Care (CSCC). These are summarised in table 1 (Tab. 1).

##### 2.1.2. Discussion phase in faculty advisory council

Next, the JUH reviewed the indicated recommendations in detail and held an open discussion in the faculty advisory council. As a result, an extraordinary, all-day session of the faculty advisory council was organised in spring 2011 on the topic of ‘Innovative Concepts for the Study of Medicine’, in which three model programmes of study and four reformed traditional study programmes were presented. A contentious discussion took place as to whether a model study programme should be created or a reformation of the current curriculum would be more sensible. 

##### 2.1.3. Political discussion phase

The Thuringian Ministry of Education, Science and Culture approved a reform of the study programme of human medicine in Jena. In particular a model study programme should be assessed against a reformed conventional curriculum. As a consequence, in the objective and performance agreement between the Science Ministry of the Federal State of Thuringia and the JUH, the introduction of a model study programme was rejected for a reform of the conventional curriculum.

#### 2.2. Objectives of academic reform

As a result of the extraordinary faculty council session in spring 2011, the academic reform was discussed intensively. In June 2011 a strengths and weaknesses analysis of the study programme of human medicine – as can be seen in table 2 (Tab. 2) – was performed. The individual academic institutions were asked to contribute initial ideas to the academic reform. Despite requests to all clinics and institutes, only a few suggestions were received (exception: student council). The objectives of academic reform in consideration of draft papers from the students were defined and united into a concept proposal by a small working group (Dean of the Faculty, Dean of Studies, Dean of Research and Representatives from the Faculty, Office of the Dean of Faculty and Office of the Dean of Studies). Special attention was devoted to early practical relevance during studies [2] and the preparation of a range of obligatory chosen modules in order to fulfil the individual needs and desired of the students [3]. 

The concept paper was adopted by the faculty advisory council in November 2011 with support from the Commission for Student Affairs (CSA, made up of the Dean of Studies, three professors, a representative of the scientific staff and two students). The concept is based on the experiences of other medical faculties but is tailored to the specific characteristics of Jena. The objectives of the academic reform and their implementation are summarised in table 3 (Tab. 3). 

#### 2.3. Structural Anchoring

After adoption of the objectives, the focus shifted to how the structural connection could be created and which content focuses the students could choose. The scope of professional interest orientation was set at 25%; the prioritisations were decided to be called tracks. After considering a connection of professional interest-oriented performance to various subject/interdisciplinary proficiency certificates, a connection to a certificate for the electives was selected. The legal office and the State Examination Board were included in the consultation on the connection to ensure that the academic reform is carried out in accordance with the law. Additional adjustments to the study and mock allocation regulations followed.

#### 2.4. Latitude for JENOS

In order to have enough space for the new electives, the curriculum in the second stage of study had to be shortened and restructured. The aim was to reduce the curriculum – with the exception of modules pertaining to bedside teaching – by 25% while conforming to the medical licence to give students comprehensive electives from which to choose. 

**Figure 1:**
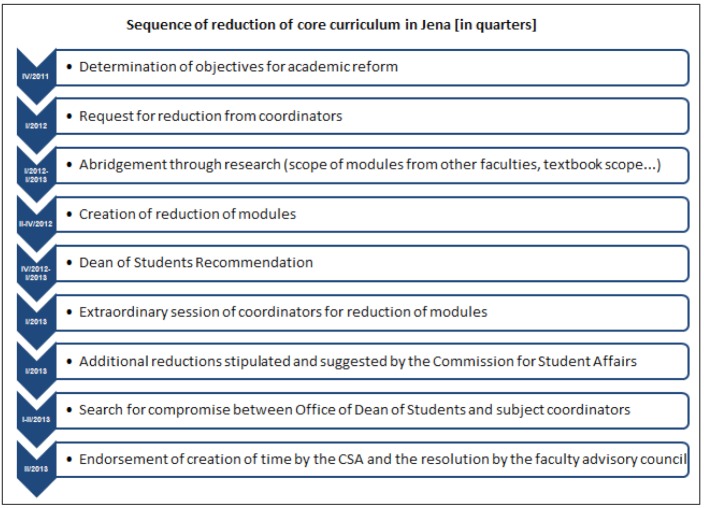
Timeline of the JENOS reform process

The faculty advisory council agreed to an abridgement of approximately 15% in July 2013 after a multi-staged process, which is described in figure 1 (Fig. 1). Table 4 (Tab. 4) describes the considered and accepted abridgement options. During this difficult process the sequence and distribution of modules in each semester was not considered initially. A sequence change could be adopted after only three months, in September 2013, by the faculty advisory council on the basis of a suggestion by the Dean of Studies. Questionnaires are summarised in table 5 (Tab. 5).

#### 2.5. Development of Electives

A challenging task was the development of electives. On one hand, they should allow students latitude to follow their own interests. On the other hand, they should also facilitate career entry through an improved practical relevance. Therefore, the following three overarching electives were agreed upon, which shall be referred to henceforth as tracks and which students must decide between: Clinic-oriented medicine (“Klinik-orientierte Medizin”; KoM), Ambulatory-oriented medicine (“Ambulant-orientierte Medizin”; AoM) and Research-oriented Medicine (Forschung-orientiere Medizin”; FoM). The prioritisation for the elective is summarised in table 6 (Tab. 6). The CSA appointed a responsible ‘Track Leader’ for creation of each of the three tracks. Working groups were created for the development of each track, typically consisting of the track leader, lecturers, students and employees from the office of the Dean of Studies. At the height of planning, the working groups met weekly to plan aims as well as the modules. The results were then presented in turn during monthly track leader meetings. The continual adjustment and optimisation process with compromises was always on the agenda. The suggestions and recommendations were then discussed again by the CSA. Surveys of students were also included in the development process. To better estimate how many students would choose each track per year, two student cohorts from each semester in the second stage of study were consulted (paper survey). This served as the foundation for calculating the capacity for each track. 

## 3. Results

The academic reform could be implemented according to plan; the first cohort began the first stage of the programme in the 2012/13 winter semester, started the second stage in the 2014/15 winter semester, took the second state examination in summer 2017, then completed the practical year and took the third state examination in 2018.

### 3.1. Objectives

In retrospect the essentials could be implemented even though not all objectives were reached. Some positives of note: a professional interest-oriented practical approach was achieved with JENOS. The integration of clinical content with regards to Introduction to Clinical Medicine (ICM) contributes to easier career entry. In the second stage of study there are at least 21 SH (=15% of the total curriculum) available for each elective (KoM, AoM, FoM) for students to take to further themselves towards their desired occupation and needs. Students are responsible for the selection of their modules, notably for those in KoM and AoM; there are no recommendations from the faculty. Depending on the motivation and choice of modules, students profit from the practical orientation while others focus more on the information provided in the module information sheet.

Additional elective modules in the core curriculum are sorted into two further topic blocks, two interdisciplinary areas and a field. Involvement of established medical specialists is successful in the AoM track: students in the AoM track choose a medical practice in which they complete a certain number of hours each semester – at least 35 teaching units (TU) in total. Before the academic reform, only contracts with general practitioners were available; after the academic reform contracts with 14 additional specialisations in a total of 70 medical practices have been signed. 

A JUH-specific lecturer and student information system (Dozenten- und Studierenden- Informations-System DOSIS), with which the schedules are created, has been in operation since winter semester 2014/15. Further optimisation of the system for planning and study organisations is needed to reduce the workload for employees. 

Modules could be reduced by approximately 25% to 1,007 TU in the second phase of study. The contents of lectures to other module formats can be found in table 7 (Tab. 7) including questions that still exist after the abridgement. Further possibilities for optimisation are possible in the area of teaching content. This will be possible to address after completion of the mapping process. 

A “Haus der Lehre” (House of Teaching) is still planned for the future. Due to the increased number of small group modules, a partial lack of space is an obstacle for offering parallel modules. The issue of the library remains unchanged.

#### 3.2. Structural Connections/Electives

The “Landesprüfungsamt” (State Examination Board) recognised electives with 21 SH in July 2013. As a result, the tracks are approximately 15% of the second phase of study; additional time could not be made available.

**Figure 2:**
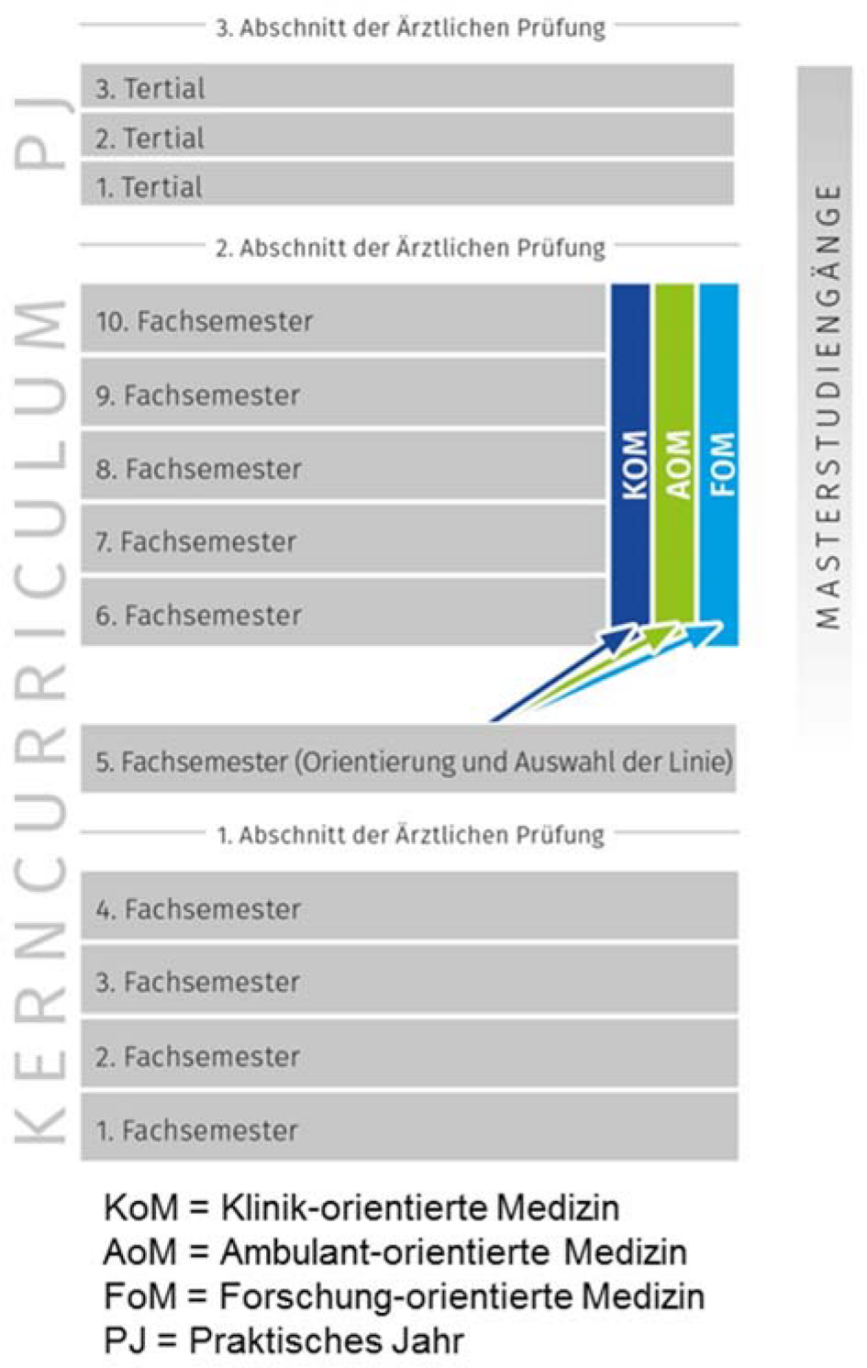
Overview of the JENOS semester set up

**Figure 3:**
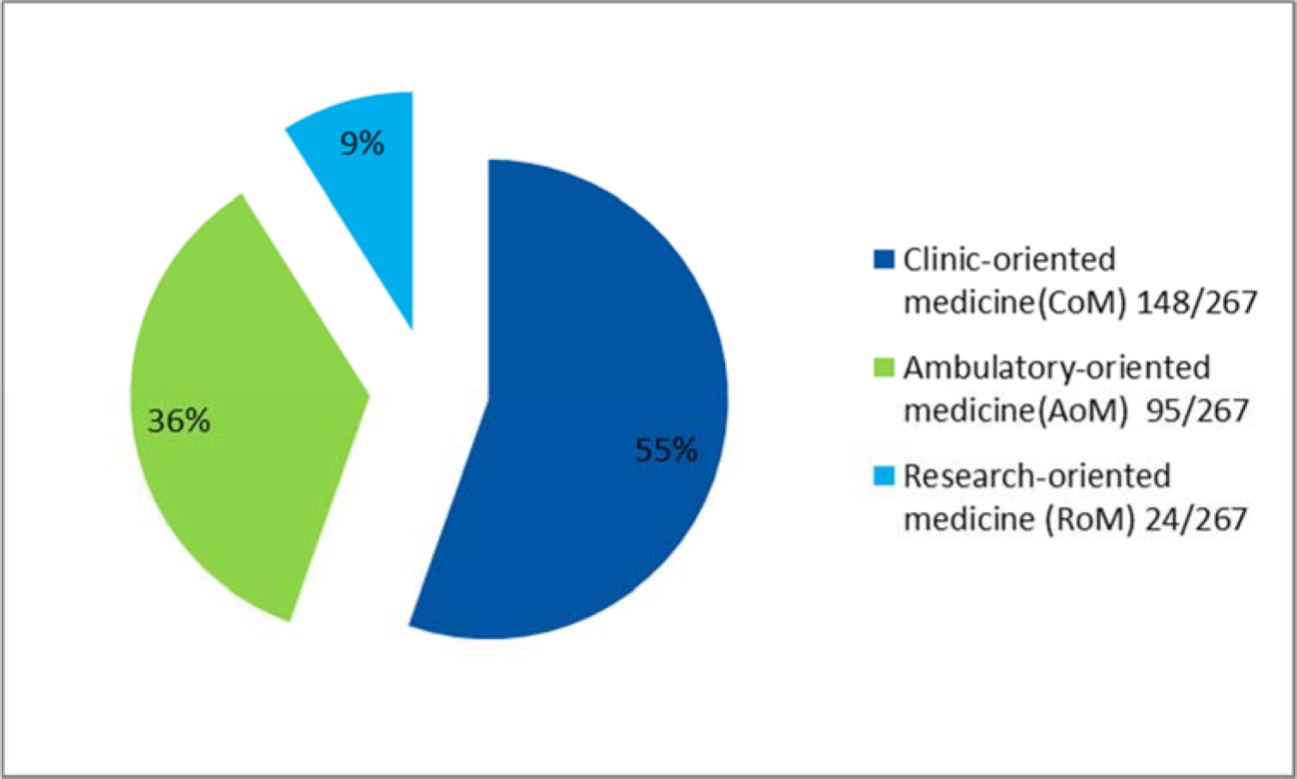
Proportion of students in each track in summer semester 2015

The election of a track occurs at the beginning of the second phase of study. Figure 2 (Fig. 2) shows an overview of the human medicine course of study. Detailed information on the tracks can be found in table 8 (Tab. 8). The distribution into three professional interest-oriented electives was first available in the 2014/2015 academic year as it appeared in the preliminary interview. The distribution can be found in figure 3 (Fig. 3). 

In the last semester of the second phase of study, an Objective Structured Clinical Examination (OSCE) occurs in order to determine the summative mark of the elective for the KoM and AoM tracks. Basic skills essential for practical career entry are tested. In addition, the students receive formative feedback. Typically for the FoM track the mark consists of a project report (60%) and its defence (40%).

#### 3.3. Feedback/Evaluations

There were initially difficulties with registering and the creation/overview of schedules when using DOSIS. The registration process for many practical-oriented modules was and is very challenging and time intensive for students. DOSIS was overloaded by the first registrations, which led to delays and complaints from the students. Many problems were resolved by increasing computing power as well as revision of the needs of the programme. 

The end-of-semester evaluations from summer semester 2015 to summer semester 2017 allowed for the recognition of significant potential for optimisation. The best evaluation was 1.2 (FoM 8th semester 2017, n=6/26, per school marking system), the worst was 3.7 (AoM, 8^th^ semester 2016, n=27/87). For evaluation results of 3.0 or worse, the organisational difficulties were identified, which are summarised in table 9 (Tab. 9). Additionally, the evaluation questionnaires were not adjusted for the modules. As of winter semester 2017/2018 evaluations of individual modules have been tested; adjustments to the questionnaire are still necessary. In addition to student surveys, lecturers have also been evaluated since the end of 2018. The analysis of the lecturer evaluation is a work-in-progress.

The assessment of JENOS from the external faculty advisory council was positive in 2016. The opportunities for students to dive into their individual interests was highlighted especially. Explicitly emphasised was the scientific orientation, which allows FoM students to purse a natural science master’s degree (e.g. Master of Molecular Medicine) and can complete a further MD/PhD programme. Although the academic reform resulted in better coupling of the first and second phases of study (e.g. using ICM), it was recommended that more clinical aspects were included in the first phase of study. 

## 4. Discussion

In order for academic reform to be successful and achieve a higher quality curriculum, not only are important reasons and a realistic schedule needed, but also the dedication of all involved actors and the availability of appropriate resources (personnel, finance, IT, rooms, etc.) are vital [7]. 

In Jena the difficulty for cost neutral academic reform was problematic; even today, some obligatory modules are not guaranteed in the long term (above all the continual administration required, teaching staff and consumable materials), which should be critiqued. Moreover the faculty only partially identified with the academic reform; it would have been easier from the beginning if all staff and students had been enthusiastic about the academic reform, leading to positive participation in the reform process [8]. This certainly works better as a primarily intrinsic motivation coming from the faculty in comparison to initiation from an outsider. To motivate the lecturers to cooperate, performance-based compensation of a maximum of € 50,000/year for five years was granted. The funds were not based on the workload. Adequate incentives could facilitate cooperation with curriculum preparation [9]. This is also important for the upcoming academic reform. With the planned changes due to the “Master Plan 2020” for Medical Studies [10], optimisation efforts of the federal government that will result in changes for the Medical Licensing Regulations for Physicians, it will be important for all participants to implement high quality changes from the beginning. 

The abridgement of modules was met with resistance. The time-consuming process at the JUH could have been better organised by a prior mapping of the learning objectives. Unfortunately, such a process could not be implemented in advance in Jena due to time constraints. The independence of teaching and therefore the subject coordinators as well as the academic chairs and their teaching staff is especially valued at the JUH. JENOS is in many areas (still) organised according to the Flexner model: those responsible for teaching determine what and how it needs to be taught and tested [11]. Essentially, the bottom-up strategy is followed in regards to teaching [1], meaning the subjects and interdisciplinary areas create their own learning objectives. This negatively influenced the academic reform as it failed to incorporate all the learning objectives of all institutions. It is still unclear how the weighting of the teaching content should be assessed. It is possible that the curriculum is influenced too much by traditional aspects or bound too tightly by structural and/or structural circumstances that have not yet been covered [1]. JENOS would benefit from better linking of learning objectives with assessments and teaching methods in accordance with Constructive Alignment [12]. This process will have to be revisited after the mapping.

A large problem within the electives was, especially at the beginning, the addition to and organisation of the range of modules. Structuring so many individual modules thematically and incorporating them well into the curriculum is a challenge to this day. It would make sense didactically if all of the students had a mentor [13], who could help them with both the selection of modules and could reflect with the students about the knowledge and skills they have acquired using e.g. a learning portfolio [12]. These factors could lead to improved learning experiences, career development and life-long learning. Those who are planning the implementation of a professional interest-oriented study programme or include additional teaching content cannot avoid the problem of making time within the existing curriculum. Recommendations as to how comprehensive subjects/interdisciplinary fields should be could be useful for similar processes. 

Although a vertical integration of clinical context was attempted in the first stage of study through the restructuring of the ICM subject, it was noted during evaluation by the faculty advisory council that further improvements were needed. A first step should be the initial obligatory chosen promotion of a longitudinal ultrasound curriculum, which begins in the first phase of study. This supports early clinical activity during studies. It can help to reduce difficulties that arise later during professional practice and also increases the motivation of students as the relation between teaching content in the earlier stage of study is illustrated through practice [1].

## 5. Conclusion

During the academic reform lecturer and student information systems were implemented in parallel. As a result, there were not only significant curricular changes but also administrative and organisational ones. At times employees faced especially challenging situations due to the changes. From the current reform process, we have learned that it is faster and more productive to spread out changes that do not need to be implemented in parallel over a wider time frame. This allows for a better implementation as well as a higher quality. 

Reforms are accompanied always by obstacles and challenges they are never easy to implement [8]. Despite the scarcity of resources and the aforementioned challenges, we were able to reform the study programme. How much work, especially how much overtime was needed by employees, is very difficult to estimate. The publication “Empfehlungen zur Weiterentwicklung des Medizinstudiums Deutschland” (Recommendations for Further Developments of Medical Studies in Germany), published in 2014, affirmed that JENOS was developed in line with the recommendations of the science council [14] and that some have already been implemented in JENOS. JENOS was used an example of positive curriculum development at the 78^th^ Ordentlichen Fakultätentag (78^th^ Annual Faculty Association Meeting) [15]. JENOS still has weaknesses and places where it can be improved. These are based on the feedback from students and the faculty advisory council. The comprehensive evaluation of students in the Master’s of Medical Education (MME) will take place in May 2019. The JUH is looking for ideas from learned experts through the MME evaluation to further optimise JENOS as well as implementation possibilities for the Master Plan 2020 for Medical Studies [10]. JENOS has already achieved some of the requirements through the academic reform in order to better implement the Master Plan. Part of the electives offered could become obligatory for all students, e.g. reinforcement of ambulatory and general medicine from modules offered in the AoM track or reinforcement of scientific character through offerings in the FoM track. To be able to plan more effectively, additional implementation details were missing despite the specifications of the Master Plan by the expert commission (published Dec. 2018) [10], [16]. There are already further requirements for implementing the Master Plan 2020. For example, curriculum mapping with the National Competency-based Catalogue of Learning Objectives for Medical Education [http://www.nklm.de] has been started for improved performance in terms of content in the next study reform. The JUH hopes that this process will make sure that all learning objectives can be mapped and targeted for weakness optimisations [17]. A top-down process for teaching content must be achieved, in which the learning objectives are defined and weighted differently at different levels and that this results in the anchoring of the content of the curriculum [1]. Therefore, it can be developed in a competence-based way which maps the entire learning process as well as learning across all stages of study [18]. For this process the JUH needs lecturers trained in medical education who are actively included in curriculum development [19]. This is the reason for attempts to expand medical didactic training in Jena and to increase the minimum scope of medical didactic programmes for those already practising. 

In order to continually improve the curriculum, it would be best to develop a good, specialised evaluation with various criteria and methods for the JUH [8]. A permanent quality management process implemented in this way is necessary in order to consistently offer high-quality teaching [7].

## Competing interests

The authors declare that they have no competing interests. 

## Figures and Tables

**Table 1:**
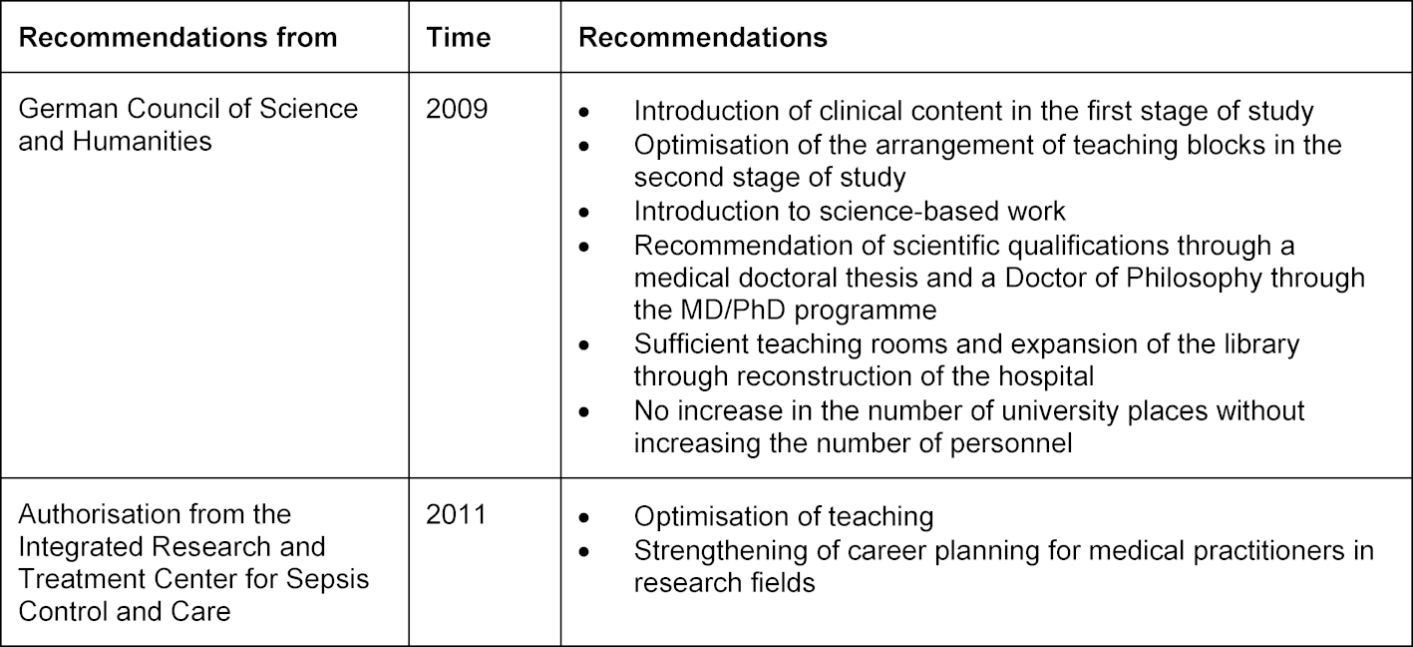
Recommendations for optimising the study of human medicine

**Table 2:**
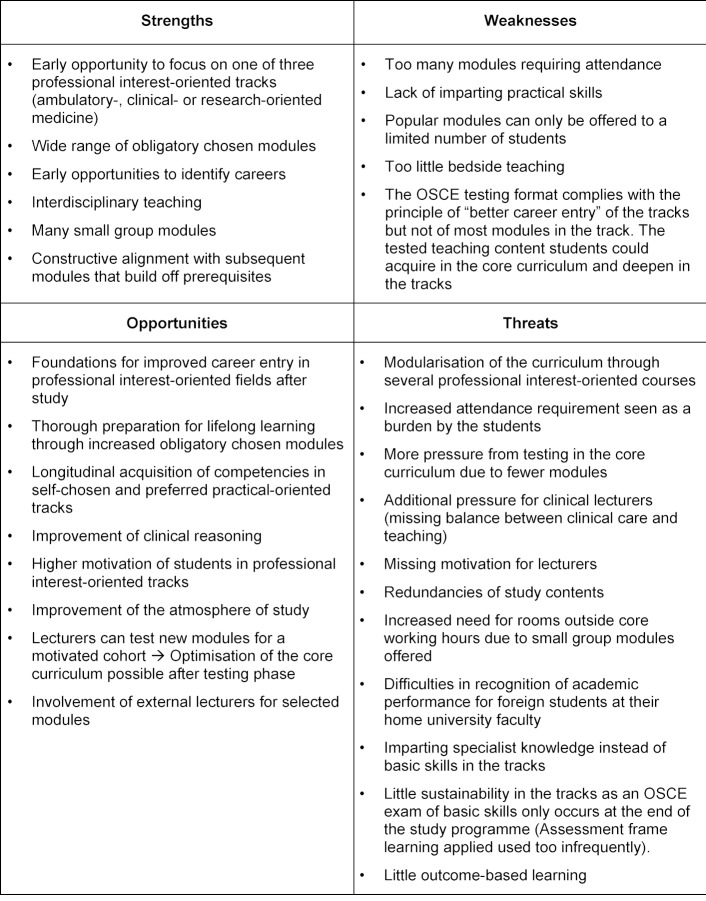
SWOT analysis of the introduction of the JENOS reform study programme

**Table 3:**
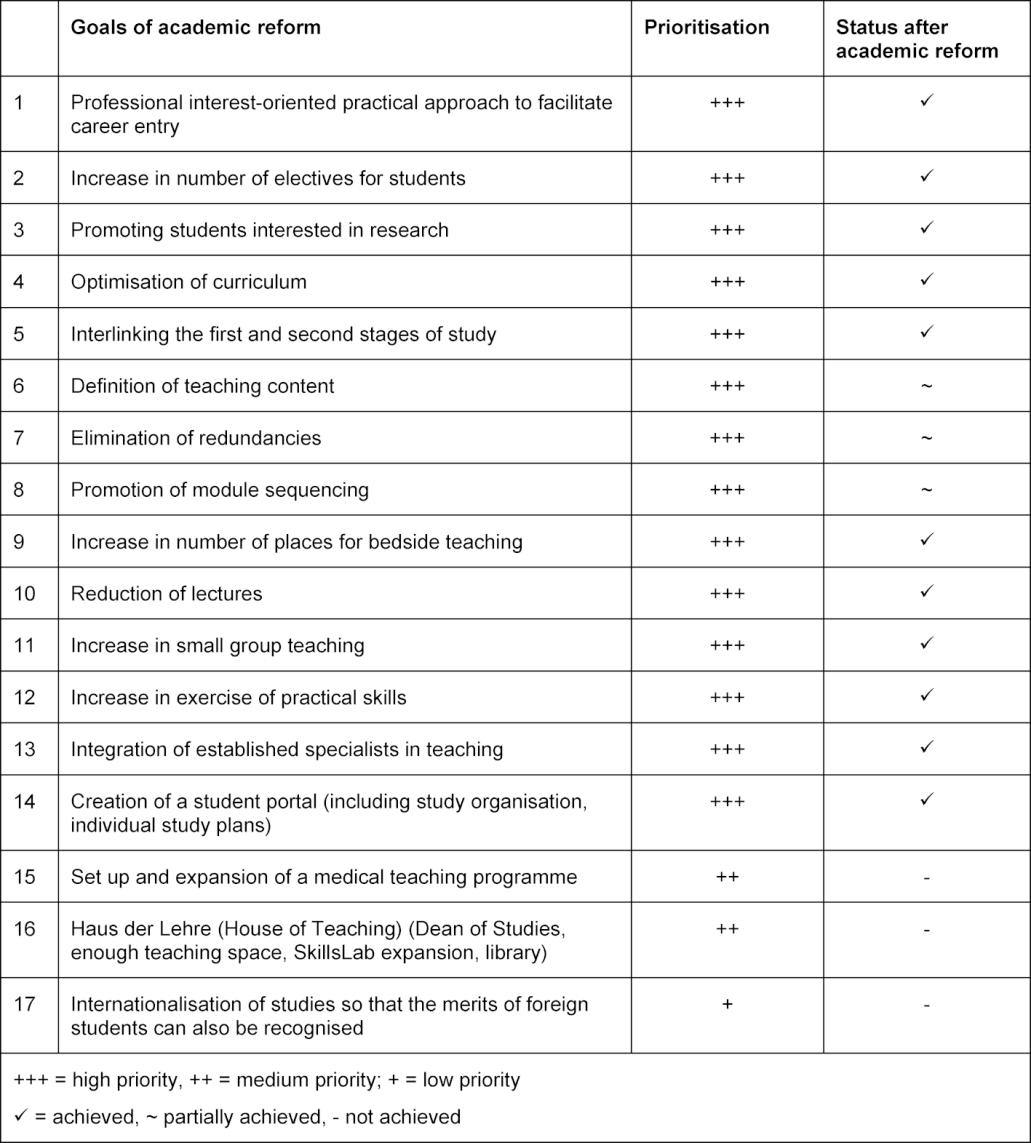
Objectives of academic reform in Jena with prioritisation and implementation

**Table 4:**
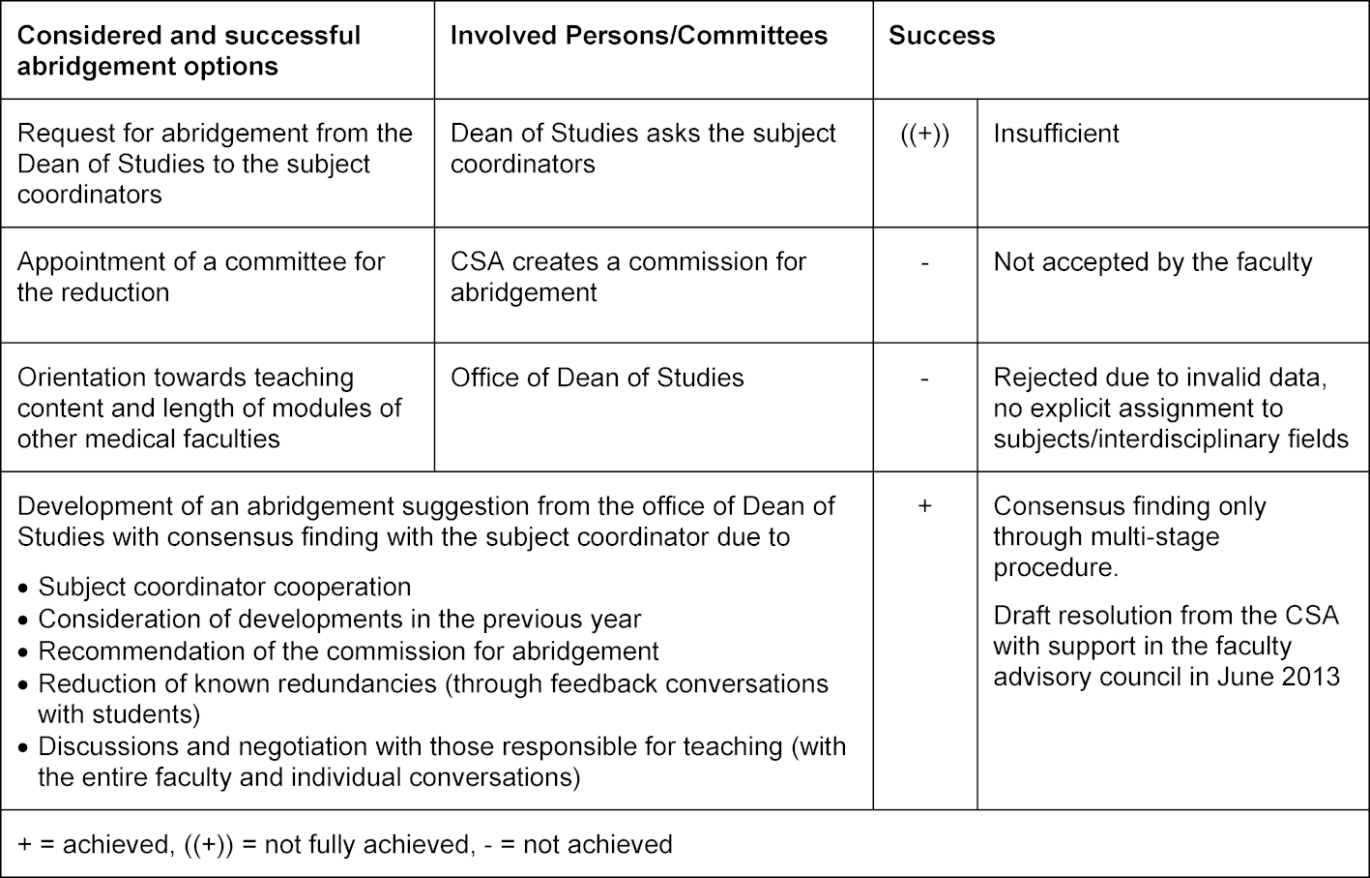
Considered and successful abridgement options

**Table 5:**
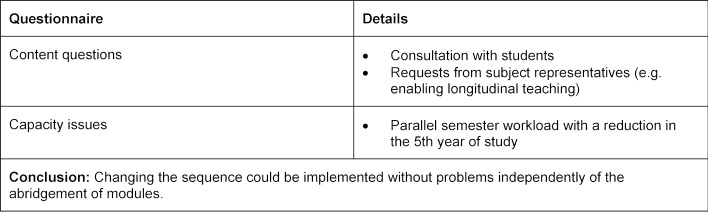
Criteria for sequence deferral

**Table 6:**
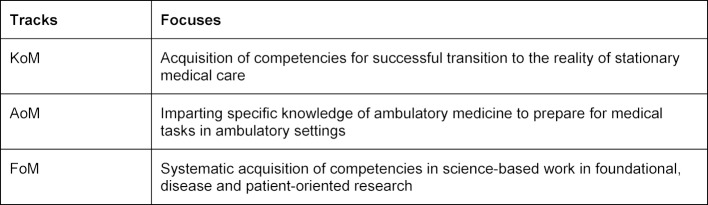
Focuses for the three electives/practical tracks: Clinic-oriented Medicine (“Klinik-orientierte Medizin; KoM), Ambulatory-oriented Medicine (“Ambulant-orientierte Medizin”; AoM), Research-oriented Medicine (“Forschung-orientiere Medizin”; FoM)

**Table 7:**
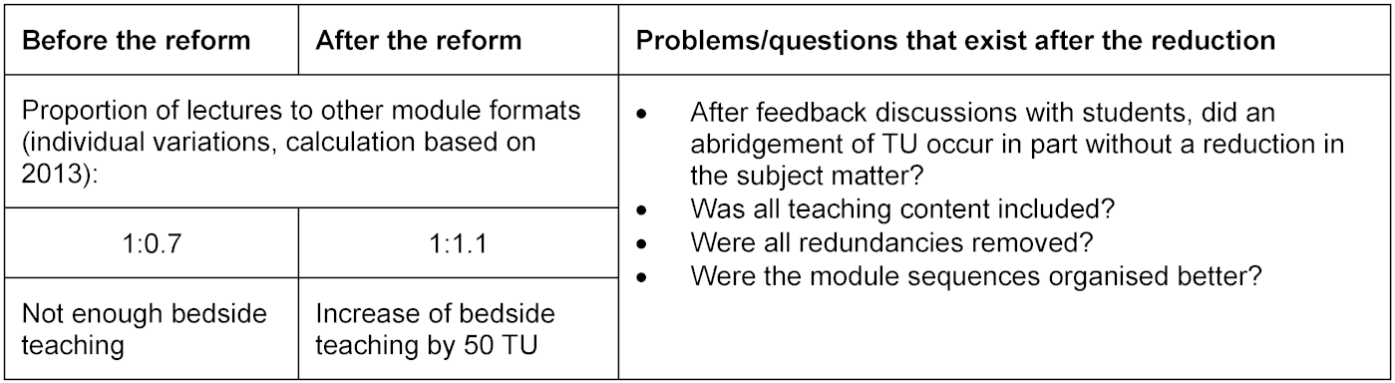
Proportion of lectures to other types of modules and questions concerning abridgement and teaching contents

**Table 8:**
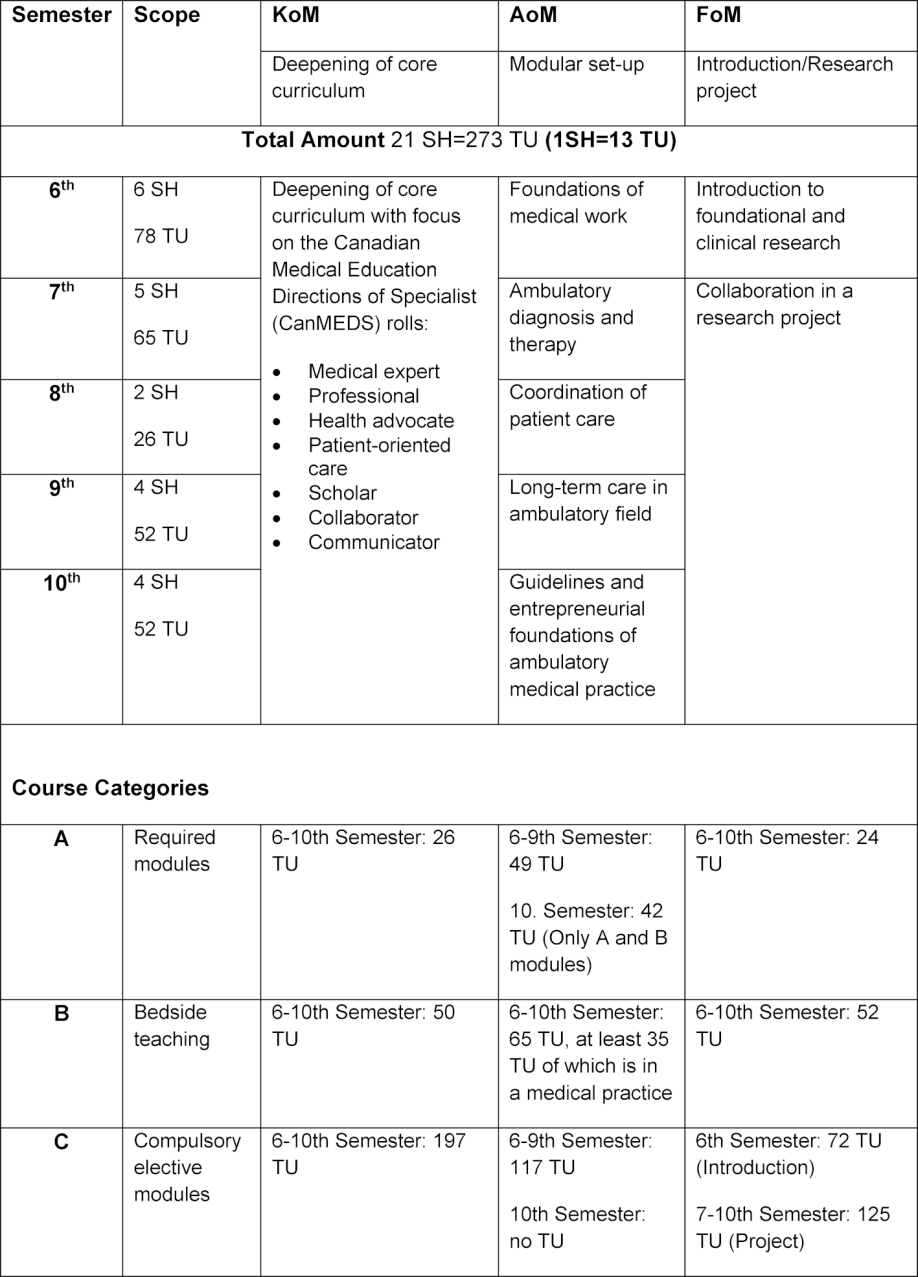
Overview of modules in the occupational tracks

**Table 9:**
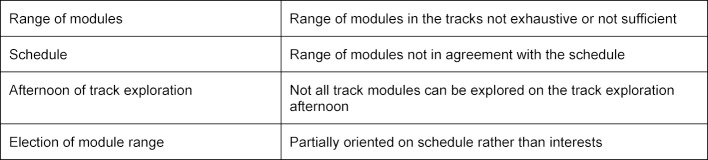
Organisational difficulties for track offers
